# Proactively restore visual function: Directly targeting affected retinal neurons

**DOI:** 10.4103/NRR.NRR-D-25-00860

**Published:** 2025-10-30

**Authors:** Ioannis Smyrnias, Ngan Pan Bennett Au

**Affiliations:** Department of Comparative Biomedical Sciences, School of Veterinary Medicine, University of Surrey, Guildford, UK

Our optic nerves are vulnerable to both traumatic and non-traumatic insults, rendering optic neuropathy a leading cause of permanent and irreversible visual impairment. Optic neuropathies can arise from hereditary [e.g., dominant optic atrophy (DOA) and Leber hereditary optic neuropathy (LHON)], ischaemic (e.g., anterior and posterior ischaemic optic neuropathy), inflammatory (e.g., optic neuritis), toxic (e.g., methanol, ethambutol) and nutritional (e.g., vitamin B12 deficiency), or traumatic conditions. Amongst these, glaucomatous optic neuropathy represents the most prevalent form and constitutes the second leading cause of blindness worldwide, with approximately 10% of patients developing bilateral blindness. Currently, over 76 million people are affected by glaucoma globally—a number predicted to rise to 112 million by 2040. Current treatments primarily focus on lowering intraocular pressure (IOP) through topical medications and surgical interventions. However, a recent study from the United Kingdom Glaucoma Treatment Study demonstrated that whilst IOP-lowering treatments effectively slow disease progression, they fail to reverse visual field deficits (Reddingius et al., 2025), underscoring the urgent need for novel therapeutic strategies that proactively restore visual function by directly targeting affected retinal neurons responsible for conveying visual information to the brain.

Permanent and irreversible visual impairment in glaucomatous and other optic neuropathies largely stems from massive neuronal loss and regeneration failure in the adult central nervous system (CNS) neurons. Following the initial insult to the optic nerve, a significant proportion of retinal ganglion cells (RGCs) initiate signalling processes for cell death alongside axonal degeneration in surviving RGCs, severing retinal–brain connections and thereby leading to decreased visual acuity and visual field defects (Borrelli et al., 2024). Unfortunately, once RGCs are lost, they cannot be naturally replaced. Additionally, surviving RGCs lack the intrinsic capacity to spontaneously regenerate the damaged axons and re-establish connections with the visual targets for functional recovery. While therapeutic strategies targeting RGC survival and neuroprotection remain important areas of investigation, stimulating regrowth of the severed axons in surviving RGCs represents another promising approach for vision restoration. Over the past two decades, strategies have been developed to enable sustained, long-distance axon regeneration following optic nerve crush (ONC) (Cartoni et al., 2016; Au et al., 2022; Liu et al., 2025). This rodent model, in which all RGC axons are severed, enables assessment of the regenerative potential of specific genetic manipulations or therapeutic interventions (Au et al., 2022). Notably, several neuroregenerative strategies not only promote axon regeneration but also lead to meaningful visual functional recovery in experimental models of glaucoma, underscoring their therapeutic potential for treating glaucomatous and other optic neuropathies.

**Figure 1:**
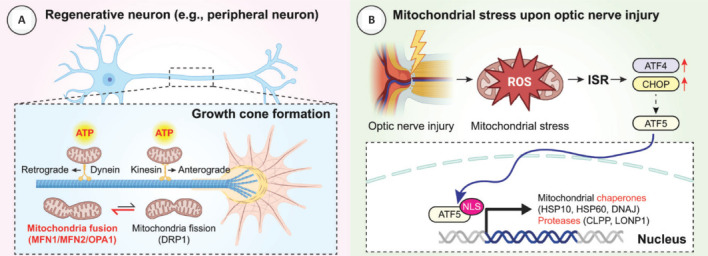
Mitochondrial dynamics are crucial for successful axon regeneration, while mitochondrial dysfunction contributes to axonal degeneration and regenerative failure. (A) In regenerative neurons (e.g., mammalian peripheral neurons or injured RGCs in zebrafish), axonal mitochondrial motility increased bidirectionally, with a pronounced redistribution of mitochondria towards distal axon tips of regenerating axons. In some cases (e.g., injured zebrafish RGCs or M1-treated peripheral neurons), enlarged mitochondrial size was observed. These enhanced mitochondrial dynamics are believed to meet elevated local energy demands, thereby facilitating the formation of growth-competent growth cones for regenerative growth. (B) Optic nerve injury triggers mitochondrial dysfunction and elevated production of ROS, activating the integrated stress response. This eventually leads to the upregulation of ATF4 and C/EBP homologous protein (CHOP), which in turn induce expression and translocation of ATF5. ATF5 functions as a key regulator of the UPR^mt^, driving transcriptional activation of genes encoding mitochondrial chaperones including heat shock proteins (HSP10, HSP60, and DNAJ) and mitochondrial proteases such as caseinolytic mitochondrial matrix peptidase chaperone subunit (CLPP) and ATP-dependent Lon peptidase (LONP1). This coordinated stress response mechanism aims to restore mitochondrial proteostasis and maintain cellular viability following optic nerve injury. Created with BioRender.com. ATF4: Activating transcription factor 4; ATF5: activating transcription factor 5; ATP: adenosine triphosphate; CHOP: C/EBP homologous protein; CLPP: caseinolytic protease P; DNAJ: DNAJ family heat shock proteins; DRP1: dynamin-related protein 1; HSP10: heat shock protein 10; HSP60: heat shock protein 60; ISR: integrated stress response; LONP1: Lon protease 1; MFN1: mitofusin 1; MFN2: mitofusin 2; OPA1: optic atrophy type 1; RGCs: retinal ganglion cells; ROS: reactive oxygen species; UPR^mt^: mitochondrial unfolded protein response.

Mitochondria are cellular powerhouses. Beyond that, mitochondria function as calcium buffers to maintain intracellular homeostasis essential for neuronal survival (Borrelli et al., 2024). Whilst most cells exist at micrometre scales where local bioenergetics adequately support metabolic demands, neurons present extraordinary challenges as highly polarised cells with specialised compartmentalisation: a compact soma connected to an axon that can extend up to 5–8 cm in human optic nerves (Borrelli et al., 2024). This creates exceptional logistical demands for maintaining constant energy supply and mitochondrial homeostasis throughout their length, which requires sophisticated mechanisms to support vital cellular functions including protein synthesis, action potential propagation, and synaptic transmission across the entire axon. To accomplish this, neurons utilise a microtubule-based motor system to facilitate both anterograde (soma-to-axon) and retrograde (axon-to-soma) transport of mitochondria along axon (**Figure 1A**). This dynamic trafficking enables strategic redistribution of mitochondria to high-demand regions (e.g., distal tip of a regenerating axon) and replacement of damaged organelles especially under stress conditions (Mar et al., 2014; Han et al., 2016; Zhou et al., 2016). Maintaining both the dynamics and functional integrity of mitochondria is therefore crucial for neuronal health.

The overall functional integrity of mitochondria is safeguarded by a suite of intrinsic mitochondrial quality control pathways to ensure the maintenance of energy homeostasis and resilience under stress. Among these, the mitochondrial unfolded protein response (UPR^mt^) has emerged as a key protective mechanism. UPR^mt^ activation engages mitochondrial-to-nuclear signalling cascades that aim to alleviate proteotoxicity and restore mitochondrial homeostasis (**Figure 1B**). However, within the CNS, where high metabolic demand is juxtaposed with limited regenerative capacity, maladaptive UPR^mt^ signalling has become increasingly implicated in neurodegenerative disorders such as Alzheimer’s and Parkinson’s diseases. Our previous work underscored the protective effects of UPR^mt^ activation induced by stress in the cardiomyocytes and overloaded heart (Smyrnias et al., 2019). Nonetheless, in RGCs, persistent activation of the integrated stress response—an effector pathway of mitochondrial dysfunction mediated via ATF4/CHOP—appears to exacerbate glaucomatous neurodegeneration. Notably, deletion of both ATF4 and CHOP in glaucomatous mouse models significantly preserved visual function (Fang et al., 2023), suggesting that rebalancing stress responses can potentially restore lost vision by protecting the retinal neurons against mitochondrial dysfunction. Given their central role in energy production, calcium homeostasis, and regulation in stress responses, mitochondrial dysfunction is unsurprisingly associated with various ocular disorders, including DOA (arising from *OPA1* gene mutations), LHON (caused by the mutations within mitochondrial DNA), as well as other neurodegenerative conditions (Borrelli et al., 2024).

**Figure 2:**
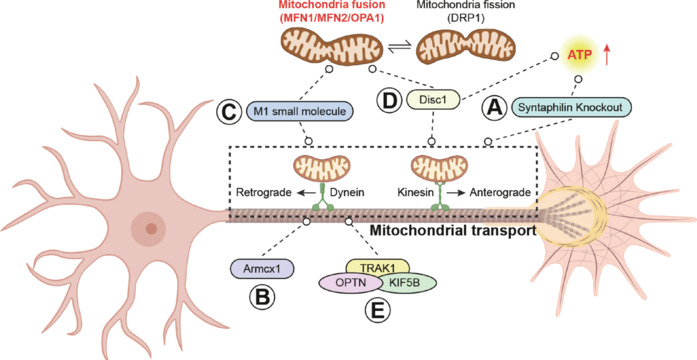
Mitochondria-targeted strategies to promote axon regeneration and restore visual function in optic neuropathy and glaucoma. (A) During neuronal maturation, progressive upregulation of the mitochondria-anchoring protein syntaphilin reduced axonal mitochondrial transport, contributing to regeneration failure in mature mammalian neurons after axotomy. Deletion of this gene restored axonal mitochondrial trafficking and ATP production post-injury, supporting growth cone formation and accelerating axon regeneration after peripheral nerve injury. (B) In injured RGCs with low regenerative capacity, the mitochondria-localised protein Armcx1 was significantly downregulated. Overexpression of Armcx1 enhanced mitochondrial motility along regenerating axons and promoted axon regeneration following ONC. (C) M1 small molecule is known as a mitochondrial fusion promoter. Treating the neurons with M1 small molecule enlarged axonal mitochondrial size via upregulation of mitochondrial fusion proteins OPA1 and MFN2, whilst simultaneously accelerated mitochondrial transport by enhancing expression of axonal mitochondrial trafficking machinery components. Remarkably, M1 treatment induced long-distance axon regeneration across the entire optic nerve length in ONC mice via OPA1- and MFN2-dependent mechanisms. This enabled the reinnervation of RGC axons to multiple visual targets for visual functional recovery. (D) In glaucomatous optic neuropathy, Disc1 downregulation caused mitochondrial fragmentation, impaired anterograde transport, and substantial reduction in ATP production. Overexpression of Disc1 restored mitochondrial size, axonal mitochondrial transport, and energy output, thereby preserving RGC functional integrity and preventing visual impairment in glaucoma mouse models. (E) Optineurin (OPTN) mutations in mice led to glaucomatous neurodegeneration by disrupting anterograde mitochondrial transport. Co-overexpression of functional OPTN with mitochondrial transport machinery KIF5B/TRAK1 restored anterograde mitochondrial transport and preserved visual function in glaucoma mouse models, as well as facilitated long-distance axon regeneration following ONC. Created with BioRender.com. Armcx1: Armadillo repeat containing X-Linked 1; ATP: adenosine triphosphate; Disc1: disrupted in Schizophrenia 1; DRP1: dynamin-related protein 1; KIF5B: kinesin family member 5B; M1: mitochondrial fusion promoter M1; MFN1: mitofusin 1; MFN2: mitofusin 2; OPA1: optic atrophy type 1; OPTN: optineurin; TRAK1: trafficking kinesin protein 1.

Axon regeneration represents an energy-demanding process, which requires continuous cytoskeletal remodelling, formation of growth-competent growth cones from severed axons, and metabolic transition from quiescent to active anabolic states to support *de novo* synthesis of cell membranes and other structural components essential for axon elongation (**Figure 1A**). Upon maturation, axonal mitochondrial transport becomes progressively compromised, contributing to age-associated decline in neuronal-intrinsic regenerative capacity (Zhou et al., 2016). Following axotomy, membrane rupture triggers excessive calcium influx at the lesion site, leading to rapid mitochondrial depolarisation and local energy deficit (Zhou et al., 2016). Peripheral nerve lesion successfully enhances axonal mitochondrial trafficking by upregulating genes involved in anterograde/retrograde transport machinery and microtubule dynamics, facilitating mitochondrial redistribution to lesion site to fulfil increased energy demands at distal axons for regenerative growth (Mar et al., 2014; **Figure 1A**). Conversely, disruption of such mitochondrial redistribution impairs axonal regrowth following axotomy (Han et al., 2016). Deletion of the mitochondrial-anchoring protein syntaphilin further boosts axonal mitochondrial motility, restores mitochondrial integrity, and resolves local energy deficits at the distal nerve stump (**Figure 2A**), thereby promoting robust axon regeneration following peripheral nerve injury (Zhou et al., 2016).

If enhancing mitochondrial dynamics can accelerate *in vivo* axon regeneration in the adult peripheral nervous system, could similar mitochondria-targeted interventions prove effective in promoting neural repair in the CNS, where axonal regrowth is virtually non-existent following injury? Interestingly, in adult zebrafish, optic nerve injury triggers redistribution of mitochondria from dendrites to axons in RGCs, accompanied by increased energy production that enables robust axonal regeneration along the optic tract into the optic tectum—the primary visual processing centre in zebrafish. During this regenerative phase, mitochondrial size increases dramatically. Nonetheless, reducing mitochondrial size in these regenerating axons by promoting Drp1-mediated mitochondrial fission impairs the regenerative process, likely due to diminished adenosine triphosphate (ATP) production by the smaller mitochondria (Beckers et al., 2023).

Given the remarkable regenerative capacity of zebrafish CNS neurons, a natural question arises: could modulating mitochondrial dynamics similarly promote optic nerve regeneration in adult mammals? Following ONC, mitochondria remain uniformly distributed along injured mammalian RGC axons (Tsuji et al., 2023), contrasting sharply with the redistribution towards distal growing tips observed in actively regenerating axons (Han et al., 2016). More concerningly, these mitochondria within the non-regenerative RGC axons are notably smaller (Tsuji et al., 2023). A recent study proposed that overexpression of Armcx1—a mitochondria-localised protein downregulated significantly in injured RGCs with poor intrinsic regenerative capacity—might counteract these injury-induced disruptions in mitochondrial dynamics. Armcx1 overexpression significantly increased axonal mitochondrial motility via interaction with the transport machinery Miro1, leading to enhanced axon regeneration following ONC (**Figure 2B**), and potentiating the regenerative effects induced by PTEN deletion (Cartoni et al., 2016). Likewise, pharmacologically promoting mitochondrial fusion with M1 small molecule not only enlarged mitochondrial size by upregulating the expression of mitochondrial fusion regulators OPA1 and MFN2, but also supported sustained, long-distance axon regeneration following ONC in OPA1- and MFN2-dependent mechanisms (**Figure 2C**). Most remarkably, M1-induced long-distance axon regeneration through the entire optic nerve length successfully restored some visual functions in ONC mice (Au et al., 2022), suggesting that targeting mitochondrial dynamics represents a potentially promising therapeutic avenue for visual restoration.

Disrupted mitochondrial dynamics has also been documented in glaucomatous optic neuropathy (Quintero et al., 2022; Liu et al., 2025). In microbead-induced ocular hypertension glaucoma models, anterograde (but not retrograde) axonal mitochondrial transport was substantially impaired in the affected optic nerve, preceding RGC loss. Additionally, mitochondrial size was markedly reduced, resulting in diminished ATP-generating capacity. This impaired mitochondrial trafficking in glaucoma was attributed to the downregulation of the mitochondrial adaptor protein Disc1 in affected RGCs and their adjacent degenerating axons. Restoring Disc1 expression via adeno-associated virus-based gene delivery to RGCs rescued mitochondrial transport deficits and restored ATP production in glaucoma mice (**Figure 2D**). More importantly, Disc1 overexpression conferred strong neuroprotective effects in those mice by promoting RGC survival, improving RGC functional integrity, and thereby preserving their visual function (Quintero et al., 2022).

A more recent study demonstrated that mutations of optineurin (OPTN), a gene known to regulate mitophagy and previously linked to normal-tension glaucoma, induced glaucomatous neurodegeneration, including progressive RGC loss and decline in visual acuity despite normal IOP levels. Dysfunction of this gene selectively depleted axonal mitochondria (i.e., decreased axonal mitochondrial density) without altering their morphology or affecting axonal transport of other cargoes, including lysosomes, endosomes, synaptic vesicles, and other cytosolic proteins. The reduced axonal mitochondrial density caused by OPTN mutation was likely driven by disrupted interaction between OPTN and the KIF5B–TRAK1 mitochondrial transport complex along axonal microtubules. Remarkably, overexpression of KIF5B–TRAK1 complex rescued mitochondrial dysfunctions and glaucomatous neurodegeneration induced by OPTN mutation (**Figure 2E**). More strikingly, overexpression of functional OPTN alongside the KIF5B-TRAK1 mitochondrial transport complex not only reversed axonal mitochondrial transport deficit and preserved visual function in an experimental glaucoma model caused by silicone oil-induced ocular hypertension, but also stimulated robust axon regeneration following ONC (Liu et al., 2025). Nonetheless, the pro-regenerative effects of Disc1 overexpression in stimulating optic nerve regeneration remain to be determined.

In conclusion, evidence accumulated over the past decade suggested that mitochondrial dynamics, including both organelle size, primarily regulated by mitochondrial fusion/fission, and motility, serves as key regulators of neuronal-intrinsic regenerative capacity and represent promising therapeutic targets for effective neural repair. Orchestrating adaptive activation of UPR^mt^, potentially via established mediators including ATF5 and HSF1 (Smyrnias et al., 2019), may confer neuroprotection by facilitating repair of damaged mitochondria, preventing apoptosis, and restoring mitochondrial homeostasis to support heightened energy demands during regenerative processes. Similarly, targeting SARM1, an enzyme that cleaves the metabolic cofactor NAD^+^ leading to mitochondrial dysfunction, calcium influx, and axonal degeneration, provided partial protection against RGC loss and visual impairment in glaucomatous mice. Nevertheless, loss of SARM1 resulted in a mild yet noticeable reduction in visual acuity even without elevated IOP (Zeng et al., 2024). Mitochondria-targeted therapies demonstrate particular relevance for inherited optic neuropathies such as DOA and LHON, where pathogenic mutations predominantly affect nuclear-encoded mitochondria-related genes (*DOA*) or mitochondrial DNA (*LHON*) (Borrelli et al., 2024). Consequently, pharmacological agents such as M1 small molecule, which rectified disrupted mitochondrial dynamics and trafficking, hold immense promise as broad-spectrum and generalised mitochondria-targeted therapeutics to foster neuroprotection and neuroregeneration across diverse forms of optic neuropathy. Additionally, leveraging bulk and single-cell RNA-sequencing datasets through publicly available resources such as the Connectivity Map or the Library of Integrated Network-Based Cellular Signatures (Au et al., 2023), alongside artificial intelligence-driven drug discovery platforms, could accelerate identification of novel mitochondria-targeted therapeutics as next-generation regenerative medicines, ultimately facilitating visual functional recovery in patients with glaucomatous and other optic neuropathies.

*This work was supported by Fight for Sight/Glaucoma UK (RESSGA2510), the Royal Society Project Grant (RG\R1\251126), and Rosetrees Trust/Stoneygate Trust (Seedcorn2024\100044) awarded to NPBA.*
